# Short‐term hypoxic training increases monocarboxylate transporter 4 and phosphofructokinase activity in Thoroughbreds

**DOI:** 10.14814/phy2.14473

**Published:** 2020-06-08

**Authors:** Wenxin Wang, Kazutaka Mukai, Kenya Takahashi, Hajime Ohmura, Toshiyuki Takahashi, Hideo Hatta, Yu Kitaoka

**Affiliations:** ^1^ Department of Human Sciences Kanagawa University Kanagawa Japan; ^2^ Equine Research Institute Japan Racing Association Tochigi Japan; ^3^ Department of Sports Sciences The University of Tokyo Tokyo Japan

**Keywords:** hypoxia, lactate, monocarboxylate transporter, skeletal muscle

## Abstract

The aim of this study was to investigate effects of short‐term hypoxic training on lactate metabolism in the gluteus medius muscle of Thoroughbreds. Using crossover design (3 months washout), eight Thoroughbred horses were trained for 2 weeks in normoxia (F_I_O_2_ = 21%) and hypoxia (F_I_O_2_ = 18%) each. They ran at 95% maximal oxygen consumption (V̇O_2max_) on a treadmill inclined at 6% for 2 min (3 days/week) measured under normoxia. Before and after each training period, all horses were subjected to an incremental exercise test (IET) under normoxia. Following the 2‐week trainings, V̇O_2max_ in IET increased significantly under both oxygen conditions. The exercise duration in IET increased significantly only after hypoxic training. The monocarboxylate transporter (MCT) 1 protein levels remained unchanged after training under both oxygen conditions, whereas MCT4 protein levels increased significantly after training in hypoxia but not after training in normoxia. Phosphofructokinase activity increased significantly only after hypoxic training, whereas cytochrome c oxidase activity increased significantly only after normoxic training. Our results suggest that hypoxic training efficiently enhances glycolytic capacity and levels of the lactate transporter protein MCT4, which facilitates lactate efflux from the skeletal muscle.

## Graphical abstract

Six sessions of hypoxic training increased the lactate transporter protein MCT4 levels and glycolytic enzyme PFK activity in equine skeletal muscle. Our results suggest that hypoxic training may be an efficient training method to enhance the glycolytic capacity of Thoroughbreds.

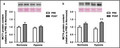

## 1 INTRODUCTION

Lactate is primarily produced in glycolytic muscle fibers during exercise and shuttled to oxidative muscle fibers as well as other organs via circulation to be used as a substrate for ATP production in mitochondria (Brooks, 2018). Lactate traverses the plasma membrane via specific transport proteins. In the skeletal muscles, monocarboxylate transporter (MCT)1 and MCT4 are known as lactate transporters, with different affinities for lactate and fiber‐type dependent distribution (Juel & Halestrap, 1999). MCT1 is a high‐affinity lactate transporter predominantly present in oxidative fibers for lactate uptake into the muscle, whereas MCT4 is a low‐affinity lactate transporter in glycolytic fibers for lactate release from the muscles (Bonen, 2001). These two MCT isoforms play a major role in the lactate shuttle in muscles during exercise. Lactate is released from glycolytic fibers via MCT4, and then taken up in oxidative fibers via MCT1 (Brooks, 2009).

Previous reports in both rodents and humans have shown that MCT1 expression increased after both continuous low‐intensity and high‐intensity training, while for increasing the expression of MCT4, high‐intensity training accompanied by an elevation of blood lactate concentration was required (Thomas, Bishop, Lambert, Mercier, & Brooks, 2012; Yoshida et al., 2004). The expression of MCT1, but not MCT4, is known to be regulated by peroxisome proliferator‐activated receptor‐γ coactivator‐1α (PGC‐1α), also called as a master regulator of genes involved in oxidative capacity (Benton et al., 2008). In contrast, hypoxia is known to activate the expression of glycolytic enzymes via hypoxia‐inducible factor 1α (HIF‐1α); MCT4, but not MCT1, is upregulated by HIF‐1α‐dependent mechanisms (Ullah, Davies, & Halestrap, 2006). We also found that high‐intensity interval training increased HIF‐1α protein level in mouse skeletal muscle (Abe et al., 2015). These previous findings led us to hypothesize that hypoxic training might be an efficient strategy to increase MCT4 protein level in skeletal muscles.

Thoroughbred horses are one of nature's most gifted athletes on the earth, with high maximal oxygen uptake (V̇O_2max_) and a large amount of glycogen in the muscles (Lacombe, Hinchcliff, Geor, & Baskin, 2001). During maximal exercise, the plasma lactate concentration reaches over 30 mmol/L, suggesting that Thoroughbreds are suitable subjects for the study of lactate metabolism (Harris, Marlin, & Snow, 1987). Indeed, we previously reported that MCT4 protein level in equine skeletal muscle was associated with exercise performance in an incremental exercise test (IET) (Kitaoka et al., 2010). We also demonstrated that high‐intensity training was required to maintain the MCT4 protein level in well‐trained horses (Kitaoka et al., 2011). A previous study reported greater improvements in running distance and V̇O_2max_ during IET after hypoxic training than normoxic training (Nagahisa, Mukai, Ohmura, Takahashi, & Miyata, 2016). However, effects of hypoxic training on skeletal muscle lactate transporters remain unknown. In this study, we examined whether 2 weeks of hypoxic training enhances exercise training‐induced adaptation of lactate transporters in equine skeletal muscle. A short‐term training period (six sessions over 2 weeks) was selected based on human studies demonstrating the rapid improvement of both anaerobic and aerobic metabolism in the skeletal muscle after only 2 weeks of sprint interval training (Burgomaster, Hughes, Heigenhauser, Bradwell, & Gibala, 2005; Rodas, Ventura, Cadefau, Cusso, & Parra, 2000).

## 2 MATERIALS AND METHODS

### 2.1 Ethical approval

All procedures were approved by the Animal Welfare and Ethics Committee of the Japan Racing Association Equine Research Institute (Permit number: 2017‐2, 2018‐2) and followed the American Physiological Society's Animal Care Guidelines. All surgeries were performed under sevoflurane anesthesia and all incisions for catheter placements were performed under local anesthesia using lidocaine. All efforts were made to minimize animal suffering.

### 2.2 Animals

Eight Thoroughbred horses (three geldings and five females) were used in this study. The horses had their carotid artery surgically moved from the carotid sheath to a subcutaneous location to facilitate arterial catheterization. After translocation of the carotid artery, horses were acclimated to running on a treadmill while wearing an open‐flow mask.

### 2.3 Experimental design

Using a randomized crossover design (3 months washout), horses were trained in normoxia (F_I_O_2_ = 21%) and hypoxia (F_I_O_2_ = 18%) 3 days/week for 2 weeks on a treadmill inclined at 6% and walked for 1 hr/day in a walker on the other 4 days for 2 weeks. Each horse was pastured in a 17 × 22 m^2^ yard for approximately 6 hr/day. The training session consisted of 1 min of cantering at 7 m/s and 2 min at the speed of 95% V̇O_2_max measured under normoxia. The duration of hypoxic stimulus per training session was approximately 5 min. Each training session was performed after warm‐up (1.7 m/s for 30 min and 4 m/s for 2 min) and followed by cool‐down (1.7 m/s for 30 min). The length of the washout period was determined based on our previous report that the protein levels of MCT1 and MCT4, as well as enzyme activities returned to the baseline levels after 6 weeks of detraining in Thoroughbreds (Kitaoka et al., 2011). Before and after each training period, the horses were subjected to incremental exercise tests (IET) under normoxia. After trotting at 4 m/s for 3 min as warm up, the horses ran on a 6% incline for 2 min each at 1.7, 4, 6, 8, 10, 12, and 13 m/s until they reached exhaustion and could not maintain their position on the treadmill. The duration of IET was measured as total time from the beginning of running at 6 m/s till the end of all‐out running. Blood samples were obtained from the catheter in a carotid artery to measure the plasma lactate concentration and hematocrit, before and at the final 30‐s of each step and 1, 3, 5 min after the IET. Blood samples were centrifuged at 12,000 *g* for 5 min to measure hematocrit, and at 1,800 *g* for 10 min to measure plasma lactate concentration using a lactate analyzer (Biosen C‐Line Glucose & Lactate Analyser; EKF‐diagnostic GmbH, Barleben, Germany). Muscle samples were taken from the same area (2 cm away from the first sampling point) at the midsection of the gluteus medius muscle and from the same depth (5 cm below the skin surface) by needle biopsy under local anesthesia (lidocaine, Fujisawa Pharmaceutical Co., Osaka, Japan) before and after each training period at rest. Muscle samples were rapidly frozen in liquid nitrogen and stored at –80°C until further analysis.

### 2.4 Oxygen consumption

Horses wore an open‐flow mask on the treadmill in which air was drawn through rheostat‐controlled blower. Air flowed through a 25‐cm diameter tube and across a pneumotachograph (LF‐150B; Vise Medical, Chiba, Japan) connected to a differential pressure transducer (TF‐5; Vise Medical). This was done to ensure that bias flows during measurements were identical to those used during calibrations. Bias flow was set to keep changes in O_2_ concentration and CO_2_ concentrations < 1.5% to keep the horses from rebreathing CO_2_. Oxygen and CO_2_ concentrations were measured with an O_2_ and CO_2_ analyzer (MG‐360; Vise Medical) and calibrations were used to calculate rates of O_2_ consumption and CO_2_ production with mass flow meters (CR‐300, Kofloc, Kyoto, Japan) using the N_2_‐dilution/CO_2_‐addition mass‐balance technique (Fedak, Rome, & Seeherman, 1981). Gas analyzer, thermohygrometer, and mass flowmeter outputs were also recorded on personal computers using commercial hardware and software (DI‐720 and Windaq Pro+, DATAQ, Akron, OH) sampling at 200 Hz.

### 2.5 Hypoxic stimulus

The procedure for producing the hypoxic condition was slightly modified from the method previously described (Ohmura et al., 2010). Briefly, a mixing chamber was connected upstream to a flexible tube on a 25‐cm diameter open‐flow mask through which N_2_ was blown into the upstream end of the flow system and mixed with a bias‐flow of air of 80–120 L/s to create the desired O_2_ concentration. Nitrogen gas flow was controlled with a mass flow meter (Model DPM3, Kofloc, Kyoto, Japan) connected to compressed gas cylinders through a gas manifold. Nitrogen gas flow was adjusted to maintain 18% O_2_ by monitoring the O_2_ concentration in the downstream arm of the mass flow meter with an O_2_ analyzer (LC‐240UW, Vise Medical, Chiba, Japan) when horses ran in hypoxia. In the second session of each training, we collected arterial blood samples in the final 30 s of galloping at 95% VO_2max_ to measure arterial oxygen saturation (SaO_2_) using (ABL800 FLEX and ABL80 FLEX‐CO‐OX, Radiometer, Copenhagen, Denmark).

### 2.6 Western blotting

Gluteus medius muscle sample was homogenized in radioimmunoprecipitation assay buffer (25 mmol/L Tris‐HCl, pH 7.6, 150 mmol/L NaCl, 1% NP‐40, 1% sodium deoxycholate, and 0.1% sodium dodecyl sulfate [SDS]) supplemented with protease inhibitor mixture (Complete Mini, ETDA‐free, Roche Applied Science, Indianapolis, IN) and phosphatase inhibitor mixture (PhosSTOP, Roche Applied Science). The total protein content of samples was quantified using the BCA protein assay (Pierce Biotechnology, Rockford, IL). Equal amounts of protein were loaded onto 12% SDS–PAGE gels and separated by electrophoresis. Proteins were transferred to polyvinylidene difluoride membranes, and western blotting was carried out, using antibodies raised in rabbits against the oligopeptide corresponding to the C‐terminal regions of equine MCT1 and MCT4 by Cosmo Bio (Tokyo, Japan), respectively. Ponceau staining was used to verify consistent loading. Blots were scanned and quantified using C‐Digit Blot Scanner (LI‐COR, Lincoln, NE).

### 2.7 Enzyme activity

Gluteus medius muscle sample was homogenized in 100 ml (v/w) of 100 mmol/L potassium phosphate buffer. Activities of two enzymes (phosphofructokinase (PFK) and cytochrome *c* oxidase (COX)) were measured spectrophotometrically to determine the glycolytic and oxidative capacities, respectively, following established protocols (Shonk & Boxer, 1964; Spinazzi, Casarin, Pertegato, Salviati, & Angelini, 2012).

### 2.8 Statistical analysis

All data were expressed as mean ± standard error of means (*SEM*). Mixed‐effects analysis followed by Bonferroni post hoc test were performed. All statistical analyses were performed by GraphPad Prism 8 (GraphPad Software, La Jolla, CA). Statistical significance was defined as *p* < .05.

## 3 RESULTS

**Figure 1:**
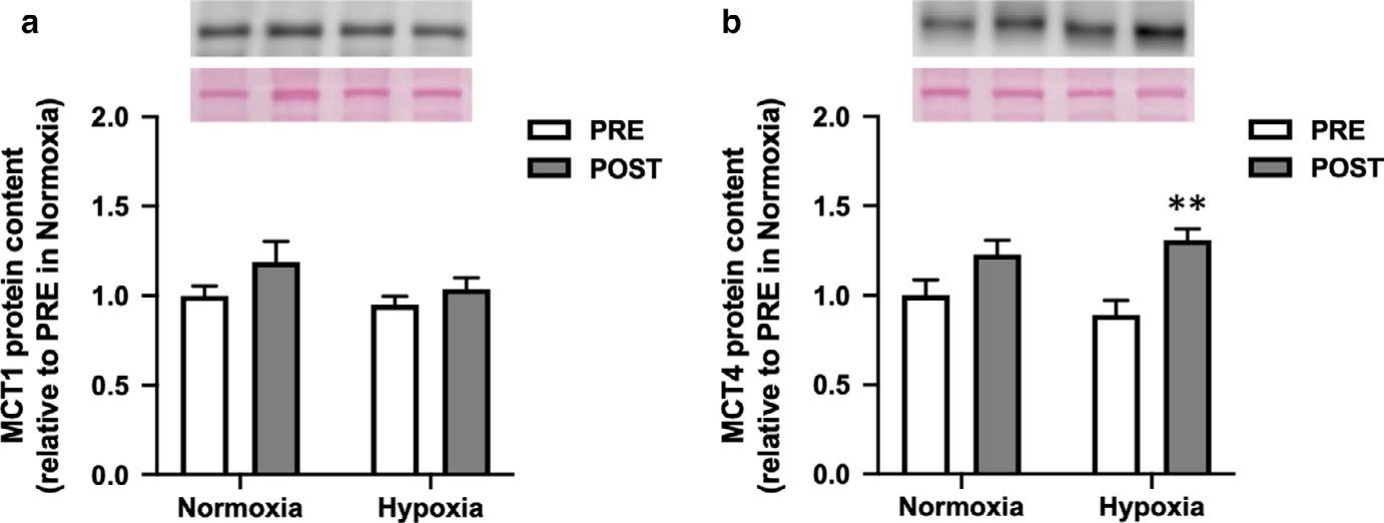
Monocarboxylate transporter protein (MCT)1 and 4 contents before (PRE) and after (POST) hypoxic training in Thoroughbred skeletal muscle. Data are presented as mean ± *SEM*. *n* = 8 in each group. **p < .01, significant difference versus pre‐training

**Figure 2:**
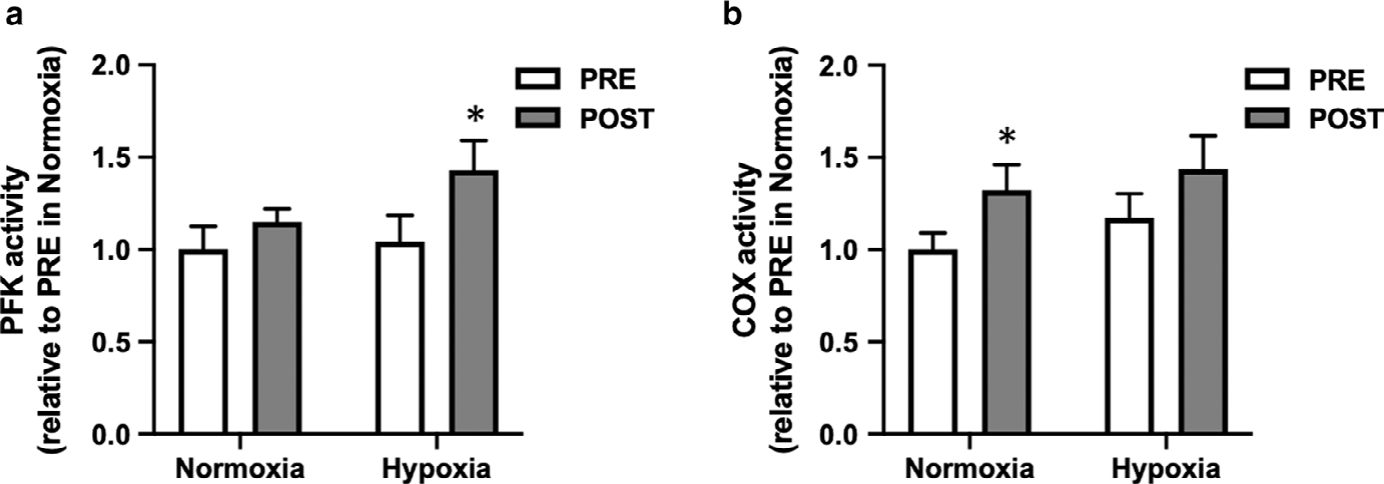
Phosphofructokinase (PFK) and cytochrome c oxidase (COX) activities before (PRE) and after (POST) hypoxic training in Thoroughbred skeletal muscle. Data are presented as mean ± *SEM*. *n* = 8 in each group. *p < .05, significant difference versus pretraining

There was no differences in pretraining values between normoxia and hypoxia for any parameters. The changes in SaO_2_ were confirmed during the second training sessions in the first week of each oxygen condition. SaO_2_ was significantly lower in hypoxia (74.3 ± 1.5%) than that in normoxia (91.0 ± 1.1%) at the end of exercise. Following 2 weeks of training under both oxygen conditions, body weight was significantly reduced, while V̇O_2max_ in the IET increased significantly (Table 1). The exercise duration increased significantly after hypoxic training and approached significance after normoxic training (*p* = .05). The plasma lactate concentration at running speed of 6 m/s in the IET was significantly decreased after training under both oxygen conditions, whereas it was significantly decreased only after training in normoxia at running speeds of 8 and 10 m/s. The lactate concentration at exhaustion in the IET was significantly increased only after hypoxic training (Table 1). There was no change in hematocrit after training under both oxygen conditions. (Table 1). MCT1 protein level remained unchanged after both trainings, although there was a trend toward significance after training in normoxia (*p* = .06, Figure 1a). MCT4 protein level increased significantly after training in hypoxia but was not altered after training in normoxia (Figure 1b). Phosphofructokinase activity increased only after hypoxic training (Figure 2a), whereas COX activity increased only after training in normoxia (Figure 2b).

**Table 1:** Body weight, maximal oxygen consumption (V̇O_2max_), peak plasma lactate concentration, and exercise duration in the incremental exercise test before (PRE) and after (POST) hypoxic training in Thoroughbreds. ^*^ *p* < .05 ^**^ *p* < .01, significant difference versus pretraining

	Normoxia		Hypoxia	
	PRE	POST	PRE	POST
Body weight (kg)	516.3 ± 8.8	505.9 ± 8.3^**^	520.8 ± 11.2	502.4 ± 10.2^**^
V̇O_2max_ (ml kg^−1^ min^−1^)	161.1 ± 3.9	173.3 ± 2.4^*^	159.0 ± 4.1	174.6 ± 2.5^**^
Exercise duration (s)	414.1 ± 24.8	446.8 ± 24.8	418.3 ± 29.1	470.9 ± 25.3^**^
Plasma lactate (mmol/L)				
Before exercise	0.9 ± 0.1	0.8 ± 0.1	0.8 ± 0.1	0.8 ± 0.1
6 m/s	2.3 ± 0.2	1.6 ± 0.1^**^	2.3 ± 0.3	1.6 ± 0.2^**^
8 m/s	5.8 ± 1.2	3.6 ± 0.5^*^	5.5 ± 1.0	3.8 ± 0.7
10 m/s	11.9 ± 1.4	8.2 ± 1.1^*^	11.2 ± 1.3	9.3 ± 1.6
Exhaustion	16.9 ± 1.7	15.1 ± 1.8	16.6 ± 1.9	18.7 ± 1.6^*^
1 min after exercise	24.3 ± 2.2	22.6 ± 1.9	23.1 ± 2.5	24.9 ± 2.3
3 min after exercise	23.4 ± 2.3	20.8 ± 2.2	22.0 ± 2.7	23.4 ± 2.4
5 min after exercise	22.2 ± 2.5	19.4 ± 2.4^*^	20.8 ± 3.0	22.1 ± 2.7
Hematocrit (%)				
Before exercise	46.3 ± 1.7	45.0 ± 2.0	45.9 ± 1.7	47.2 ± 1.9
6 m/s	52.8 ± 1.0	52.4 ± 1.2	54.2 ± 1.1	55.0 ± 1.4
8 m/s	57.1 ± 1.3	56.4 ± 1.4	58.2 ± 0.9	58.9 ± 1.0
10 m/s	60.4 ± 1.2	59.4 ± 0.9	60.7 ± 1.0	61.3 ± 1.0
Exhaustion	61.4 ± 1.1	60.1 ± 1.1	61.0 ± 1.1	62.8 ± 1.1
1 min after exercise	62.8 ± 1.3	63.3 ± 1.1	63.6 ± 1.2	64.3 ± 1.2
3 min after exercise	61.9 ± 1.4	62.6 ± 1.2	62.0 ± 1.2	63.2 ± 1.4
5 min after exercise	60.8 ± 1.4	60.9 ± 1.2	60.8 ± 1.3	62.1 ± 1.4

## 4 DISCUSSION

Hypoxic training has been utilized as a method to improve athletic performance in human athletes. The underlying mechanisms of exercise‐based performance improvement are considered to lie mainly in cardiovascular and hematological adaptations (Viscor et al., 2018). In Thoroughbreds, hypoxic training is reported to improve exercise performance, associated with the increase in V̇O_2max_ but without changes in packed cell volume, suggesting the contribution of non‐hematological adaptations (Nagahisa et al., 2016; Ohmura, Mukai, Takahashi, Takahashi, & Jones, 2017). However, it is still unknown whether hypoxic exposure during training leads to greater changes in muscular metabolic characteristics. The major finding of this study showed that short‐term training in hypoxia enhances glycolytic enzyme activity and lactate transporter MCT4 protein levels in Thoroughbred skeletal muscles.

### 4.1 Monocarboxylate transporters

In human studies, it has been well established that high‐intensity interval training increases both MCT1 and MCT4 protein levels in skeletal muscles (Burgomaster et al., 2007; Perry, Heigenhauser, Bonen, & Spriet, 2008). In accordance with these reports in humans, we previously demonstrated that high‐intensity training increased MCT1 and MCT4 protein levels in Thoroughbred horses. Interestingly, the increase in MCT1, but not MCT4, was maintained with moderate‐intensity training (Kitaoka et al., 2011). In this study, we found that hypoxic training increased MCT4 protein expression, suggesting that training in hypoxia could be more effective than similar training in normoxia in Thoroughbreds. This finding is in line with an earlier report showing an upregulation of MCT4 mRNA expression after intermittent hypoxic training (all‐out repeated sprints) in trained cyclists (Faiss et al., 2013). However, although MCT4 was shown to be up‐regulated by severe hypoxia (Py et al., 2005; Ullah et al., 2006), previous studies reported that there was no change in MCT1 and MCT4 after acclimation to high altitude (Juel, Lundby, Sander, Calbet, & Hall, 2003), and “live high, train low” hypoxic exposure (Clark et al., 2004) in humans. The lack of changes in lactate transporters in these reports suggested that more intense training may be required under hypoxic conditions.

### 4.2 Glycolytic capacity

In Thoroughbred horses, the activity of glycolytic enzymes generally do not change following training (Cutmore, Snow, & Newsholme, 1985; Serrano, Quiroz‐Rothe, & Rivero, 2000). Unlike humans (MacDougall et al., 1998; Rodas et al., 2000), even high‐intensity training does not enhance PFK activity in Thoroughbreds (Kitaoka et al., 2011), with few exceptions as examined in long‐term training effect over 3 months (Eto et al., 2004). This was thought to be due to the inborn high glycolytic capacity of equine skeletal muscles. However, here, we found that six sessions of hypoxic training increased PFK activity, suggesting that there is trainability for glycolytic capacity in Thoroughbreds. Our finding of increased PFK activity after hypoxic training is consistent with several previous reports in mice (Suzuki, 2019) and humans (Puype, Van Proeyen, Raymackers, Deldicque, & Hespel, 2013). Moreover, exercise‐intensity–dependent changes in PFK mRNA was augmented by hypoxia in human skeletal muscle (Vogt et al., 2001; Zoll et al., 2006). Taken together, the addition of hypoxic stress to exercise training augmented glycolytic capacity in the skeletal muscle of Thoroughbreds.

### 4.3 Oxidative capacity

In contrast to glycolytic enzymes, it has been demonstrated that long‐term exposure to hypoxic conditions leads to a decrease in muscle mitochondrial enzyme activities, including citrate synthase (CS), succinate dehydrogenase (SDH), and COX (Green, Sutton, Cymerman, Young, & Houston, 1989; Howald et al., 1990; Levett et al., 2012). Proteomic analysis showed that chronic exposure to hypoxia induced downregulation of proteins involved in tricarboxylic acid cycle and electron transport, and upregulation of glycolytic enzymes in rat skeletal muscles (De Palma et al., 2007). The addition of hypoxic stress during exercise training has been reported to additively increase mRNA levels but do not affect activity of oxidative enzymes (CS and COX) in trained human skeletal muscles (Zoll et al., 2006). Another study in untrained subjects showed that training in hypoxia resulted in greater increase in CS activity but not SDH activity, compared with the same amount of training in normoxia (Melissa, MacDougall, Tarnopolsky, Cipriano, & Green, 1997). Likewise, training in hypoxia with the same relative workload as in normoxia had similar effects on muscle mitochondrial volume in humans (Desplanches et al., 1993). In this study, we also found that there was no additional effect of the hypoxic stimulus on training‐induced COX activity in Thoroughbreds. This result was consistent with a previous study demonstrating that training‐induced increase in muscle oxidative function observed during normoxia was absent during hypoxia (Bakkman, Sahlin, Holmberg, & Tonkonogi, 2007). However, it is worth noting that hypoxic training may alter the intrinsic properties of mitochondrial function without changes in oxidative enzyme activity (Ponsot et al., 2006; Roels et al., 2007).

### 4.4 Potential mechanisms

The cellular response to hypoxia is mainly mediated by HIF‐1, which is identified as master transcription factor of oxygen homeostasis (Wang & Semenza, 1995), and therefore, HIF‐1α has been recognized as the key mediator of adaptations to hypoxic training. Several studies have reported that hypoxic training increased mRNA levels of HIF‐1α in the skeletal muscle of humans (Vogt et al., 2001; Zoll et al., 2006) and Thoroughbreds (Nagahisa et al., 2016). Given that pharmacological activation of HIF‐1α elevated mRNA expression of PFK and MCT4 (Abe et al., 2015), our results of increased PFK activity and MCT4 protein level in the current study might be partly owing to the activation of HIF‐1α pathway by hypoxic training. While HIF‐1α regulates glycolytic metabolism, PGC‐1α is a potent regulator of oxidative metabolism and MCT1 in the skeletal muscle (Benton et al., 2008; Lin et al., 2002). It is well established that high‐intensity training increases PGC‐1α protein level in the skeletal muscle of humans (Little, Safdar, Wilkin, Tarnopolsky, & Gibala, 2010) and horses (Kitaoka et al., 2012). Importantly, high‐intensity exercise‐induced PGC‐1α mRNAs were attenuated under hypoxia in Thoroughbred skeletal muscle (Okabe, Mukai, Ohmura, Takahashi, & Miyata, 2017). This hypoxia‐induced reduction of PGC‐1α might diminish the mitochondrial adaptation to exercise training.

### 4.5 Lactate metabolism during exercise

Increased PFK activity and MCT4 protein level by hypoxic training reveal enhanced capacity for anaerobic ATP production. Given that the transport of lactate via MCT is coupled with the transport of a proton, increased MCT4 might play an important role in maintaining intramuscular pH (Juel & Halestrap, 1999), which prevents the low pH induced inhibition of glycogenolysis during exercise (Hollidge‐Horvat, Parolin, Wong, Jones, & Heigenhauser, 1999). Supporting this notion, we previously found a correlation between MCT4 protein content and muscle lactate concentration immediately after 2 min of intense exercise at 120% VO_2max_ in Thoroughbreds (Kitaoka et al., 2014). In the present study, the plasma lactate concentration at exhaustion during IET was higher after hypoxic training than normoxic training, although exercise performance in the IET increased to a similar extent after both training (7.9% in normoxia and 12.6% in hypoxia). A previous study reported that MCT4 protein content was correlated with average power output during the 2‐min time trial, but not during 10‐min time trial in trained cyclists (Bentley et al., 2009). These observations suggested that MCT4 becomes more important during short and intense exercises that require very high rate of glycolytic ATP production. Thus, we speculated that the duration and form of the IET might have masked the effects of enhanced glycolytic capacity, since it depended more on oxidative metabolism, which was not altered by hypoxic training. In contrast to the values at exhaustion, the plasma lactate concentration at submaximal intensity during IET decreased following hypoxic training, despite that MCT1 protein level and mitochondrial enzyme activity were not altered. Increased capillary density might have contributed in increased lactate uptake in oxidative tissues, since it was previously observed only after hypoxic training in Thoroughbreds (Nagahisa et al., 2016).

Lastly, a limitation of the current study is its relatively small sample size. In a larger study using 163 healthy Thoroughbreds, the proportion of Type IIB fibers is reported to be different between sexes; however, importantly, no differences were seen in metabolic enzyme activities between sexes (Roneus, Lindholm, & Asheim, 1991). Considering that we did not observe any differences in enzyme activities and MCT protein levels between sexes in our previous studies in Thoroughbreds (Kitaoka et al., 2011, 2012), we included horses of both genders in this study. In addition, it should be noted that enzyme activity does not necessarily represent the actual metabolic flux in vivo. Further studies are required to elucidate the effects of hypoxic training for a longer period on lactate metabolism during exercise.

## 5 CONCLUSION

In this study, we examined the effects of short‐term hypoxic training on MCT and indicators of energy metabolism in the gluteus medius muscle of Thoroughbreds. We demonstrated that training in hypoxia enhances glycolytic enzyme activity and MCT4 protein level but has no additional effects on oxidative enzyme activity and MCT1 protein level in Thoroughbred skeletal muscle.

## CONFLICTS OF INTEREST

None of the authors has any conflict of interest to disclose.

## AUTHOR CONTRIBUTIONS

K.M., H.H., and Y.K. conceived and designed the experiments. K.M., H.O., and T.T. collected samples. W.W., K.M., K.T., and Y.K. performed experiments. W.W. and Y.K. analyzed data and interpreted results of experiments. W.W. and Y.K. drafted the manuscript. All authors revised and approved the final version of this manuscript.
